# Synchronous Mindfulness in Motion Online: Strong Results, Strong Attendance at a Critical Time for Health Care Professionals (HCPs) in the COVID Era

**DOI:** 10.3389/fpsyg.2021.725810

**Published:** 2021-08-18

**Authors:** Maryanna Klatt, Rani Bawa, Olivia Gabram, Alexis Westrick, Amanda Blake

**Affiliations:** ^1^Department of Family and Community Medicine, College of Medicine, The Ohio State University, Columbus, OH, United States; ^2^Wexner Medical Center, The Ohio State University, Columbus, OH, United States

**Keywords:** mindfulness, mindfulness in motion, virtual delivery, COVID-19, healthcare professional, synchronous, online mindfulness intervention, attendance

## Abstract

Mindfulness in Motion (MIM) is an organizationally-sponsored mindfulness program for employees at a large academic health center that consistently produces significant reductions in burnout and perceived stress, alongside significant increases in work engagement and resilience. This study compared outcome measures of a synchronous virtual delivery of MIM, necessitated by COVID-19, to traditional in-person delivery of MIM. Outcome measures from the virtual COVID (AU20, WI21, SP21) MIM cohorts (*n* = 99) were compared with the in-person Pre-COVID (SP19, AU19, WI20) MIM cohorts (*n* = 124). Both Pre-COVID and COVID cohorts had similar attendance rates with an average attendance of 84 and 80%, respectively. Qualitative analysis of COVID cohorts reported community support during COVID as a substantial intervention benefit, which was important at a time when isolation dominated the healthcare professional experience. Total burnout was determined by scores on the subscales of the Maslach Burnout Inventory (MBI). There were no significant differences in depersonalization (*p* = 0.3876) and personal accomplishment (*p* = 0.1519) changes between Pre-COVID and COVID cohorts, however there was a significant difference in emotional exhaustion (*p* = 0.0315), with COVID cohorts improving more. In both Pre, and COVID cohorts, the percentage of people meeting burnout criteria from pre to post between groups were similar, yielding a non-significant difference (*p* = 0.2950). The Connor Davidson Resiliency Scale (CDRS) and Utrecht Work Engagement Scale (UWES) also produced no significant differences between groups (*p* = 0.4259, *p* = 0.1984, respectively). The Perceived Stress Scale (PSS) though yielded significant differences in reduction between groups (*p* = 0.0405), again with COVID cohorts showing greater improvement. Results of the first synchronous, virtually delivered MIM cohorts reflect that participants achieved very similar results and that MIM created a community in a time when it was greatly needed due to pandemic healthcare professional stress.

## Introduction

Healthcare Professionals (HCPs) face a variety of workplace stressors that contribute to high rates of burnout (Dyrbye et al., 2017). There is a rich body of research detailing the deleterious effects of burnt-out HCPs, including but not limited to compromised patient safety, lessened cost-effectiveness, and high turnover within a health system (West et al., 2018). However, one avenue that has proven effective in reducing burnout among HCPs is mindfulness. Mindfulness-based interventions successfully reduce burnout among HCPs, leading to downstream benefits affecting HCPs themselves, the patients they care for, and the health system who employs them (Luken and Sammons, 2016). Mindfulness in Motion (MIM), one such mindfulness-based intervention designed specifically for HCPs, has been studied in detail with strong results showing burnout reductions (Klatt et al., 2015, 2018, 2021; Steinberg et al., 2017; Moffatt-Bruce et al., 2019; Klatt M. D. et al., 2020). Until now, this program, like most research surrounding mindfulness-based interventions for HCPs, has focused on in-person interventions, though other virtual mindfulness-based interventions have been studied and reported in the literature.

### Existing Virtual Mindfulness-Based Interventions

Research has shown that virtual mindfulness-based interventions are feasible and effective, yet the existing literature is sparse (Jayawardene et al., 2017). While in-person mindfulness-based interventions have been extensively studied, yielding a host of benefits, virtual mindfulness-based interventions have the potential to extend these benefits during times when in-person programming is not possible, such as during a pandemic. A study (*n* = 89) examining the impact of online workplace mindfulness interventions on employee stress, resilience, and well-being found that the mindfulness intervention significantly decreased perceived stress while increasing resilience (Aikens et al., 2014). In a large study of *n* = 219 participants, results of a public online mindfulness program indicated a dose-dependent relationship between number of days mindfulness was practiced and amount of improvement among perceived stress, affect, flourishing, and self-compassion measures (Bailey et al., 2018). A recent meta-analysis combining the results of eight randomized controlled trials of online mindfulness interventions showed that significant effects were found for reductions in perceived stress following the interventions. The authors noted, however, the lack of existing studies on online mindfulness, warranting the present study (Jayawardene et al., 2017). In addition, a strength of online interventions is that they have the ability to reach participants who may be even more in need of mindfulness programming and are unable to attend in-person programming (Bailey et al., 2018).

### COVID-19 Impacts on HCPs

At the start of COVID-19 pandemic in the United States, the authors realized that HCPs now, more than ever, needed workplace mindfulness programming (Shanafelt et al., 2020; Loucks et al., 2021). However, they were faced with a novel challenge: how to provide mindfulness programming to frontline workers without the ability to be in a room together, as even hybrid delivery of MIM involved participants and facilitators in the same physical space. Using the framework of transitioning from creator-led to hybrid delivery of MIM (Klatt M. et al., 2020), the same process was undertaken to transition from hybrid delivery to fully virtual delivery. Though MIM is a secular program, it helps participants learn how to incorporate mindfulness into their existing spirituality if desired. Research shows that spirituality can be an important part of healthcare (Chirico and Magnavita, 2019), as well as that spirituality can be integral in coping with COVID-19 stressors and increased burnout rates faced by HCPs (Chirico, 2021; Chirico et al., 2021; Magnavita et al., 2021). There were specific elements of the transition from creator-led to hybrid delivery that were replicated and maintained in the transition from hybrid delivery to fully virtual delivery that allowed for a seamless transition: the fidelity monitoring system discussed below, and the weekly meetings between facilitators, fidelity checkers, program creator, and program manager.

### Fidelity Monitoring System

The fidelity monitoring system was necessary for both the transition from creator-led to hybrid delivery as well as the transition from hybrid delivery to fully virtual delivery of MIM. Without a fidelity checking system, the internal validity of the results were at risk. Because internal validity was established, dissemination could be expanded in order to measure external validity to see if results are sustainable for over a year after the end of the 8-week program (Klatt et al., 2021). Analyses show that the decreases in perceived stress and burnout, as well as the increase in resilience, were still significantly improved between pre and follow-up time points, with the average follow-up time being 12.2 months. The results for work engagement were not significant, though still trended in the positive direction (Klatt et al., 2021).

### Weekly Meetings

In addition to establishing results with strong internal and external validity, weekly meetings were held between facilitators, fidelity checkers, and the creator of MIM. These weekly meetings helped to make sure all fidelity checkers and facilitators were updated of any changes that arose as well as allowed any problems to be solved as a team. Internet connectivity issues, Zoom platform troubles, and retention were discussed with solutions generated in these meetings. Not only did these meetings help ensure consistency across facilitators and cohorts, but also helped to address issues related to virtual delivery in a timely manner.

The literature reviewed shows that virtual delivery of mindfulness programs is feasible, but it was unknown whether or not virtual delivery of MIM replicated the results of in-person delivery of MIM. The present study aims to compare Pre-COVID, in-person delivery of MIM to COVID, fully virtual delivery of MIM on four main outcome measures: perceived stress, burnout, resilience, and work engagement. This addresses a recent call in the literature to compare effectiveness of virtual mindfulness interventions to in-person interventions containing the same content (Jayawardene et al., 2017).

## Methods

Mindfulness in Motion (MIM) is an evidence-based, workplace mindfulness-based intervention effective in reducing perceived stress and burnout while increasing work engagement and resilience (Klatt et al., 2015, 2017, 2018). The study is a non-randomized, single arm, pre/post study with Institutional Review Board approval from The Ohio State University Institutional Review Board (Study #2017B0321). This program now exists in three delivery modes: creator-led, hybrid delivery, and virtual delivery. This manuscript details each of these modes with a focus on virtual delivery necessitated by the COVID-19 pandemic.

Inclusion criteria for the study were: being an employee of The Ohio State University Wexner Medical Center, being 18 years or older, and having access to the internet to complete website-based home practices. Exclusion criteria was the inability to speak and understand English, as the program is run in English. Consent was obtained from participants of MIM to be a part of this research study. Analysis was completed on participants who consented to have their data analyzed in the study as well as completed a full pre and post data set via an institutional password protected website.

Beginning with a pilot study of MIM with 32 participants from a surgical intensive care unit (Duchemin et al., 2015), the program has now reached over 420 healthcare professionals (HCPs). While initially, the size and scale of the program was manageable for the first author to deliver on her own, the demand for the program quickly outgrew her individual reach. In response to demand, a facilitator training model was developed and videos were filmed to create a new delivery mechanism of MIM: hybrid delivery (Klatt M. et al., 2020). The hybrid delivery model of MIM utilized video recordings of the creator explaining the science behind mindfulness (didactic video) as well as guiding participants in an experiential mindfulness meditation and gentle yoga session (experiential video). The trained facilitators led the discussion portion of the program and showed the didactic and experiential videos, following a detailed protocol contained in their standardized training manual to ensure consistency. Comparing the two delivery methods, results show no significant differences across outcome measures of perceived stress, burnout, work engagement, and resilience, supporting the validity of the hybrid delivery model of MIM (Klatt M. et al., 2020).

A key development that emerged during the transition to hybrid delivery was the need for a fidelity monitoring system to ensure that the intervention protocol was the same as creator-led MIM. There was a 98.86% adherence rate to the protocol, as determined by a detailed fidelity monitoring system developed for MIM, involving a fidelity checker (a research assistant) present for each session. This fidelity monitoring system was operational for hybrid delivery MIM before the COVID-19 pandemic, helping to create a seamless transition from hybrid delivery to fully virtual delivery of MIM.

There are multiple elements of the MIM program that needed reconfiguration in order to accommodate a fully virtual delivery mode (see Table 1). The most important elements are described in detail below. An essential pivot to transition to a fully virtual format was changing the format, not the content, of the program materials. Traditionally, a workbook is issued on week one to participants to accompany the class work each week, however, if meeting online, hardcopy versions could not be administered. Thus, the workbook was adapted to online documents that were emailed to participants each week prior to class. Participants were urged to save these worksheets as they go through the 8-weeks for their own personal use. Furthermore, the workbook contains a tracking sheet for pre/post breath counts that are conducted each week to determine participant respiratory rate trends. To gather this data, secure Qualtrics survey links were sent into the zoom chat for participants to log their pre/post breath count each week and the tracking sheet of participant daily practice was emailed to each participant. An existing structural component of MIM that made the transition to fully virtual programming feasible was the website. The secure, password protected MIM website contains home practices, module questions and further readings to help encourage a mindfulness practice. Retaining this on a virtual platform was essential for a seamless transition.

**Table 1:** Changes made to accommodate virtual delivery of MIM.

**Element of program**	**Creator-led delivery**	**Hybrid delivery**	**Fully virtual delivery**
IT training	N/A	Facilitators trained to show didactic and experiential videos through smart-screen	Facilitators and fidelity checkers trained in digital accessibility and to use Zoom in order to share didactic and experiential videos, send links in chat, and share music
Workbook	Paper workbook handed out on first day of program		Relevant workbook pages sent out electronically weekly rather than all at once, including daily tracking of practice
Didactic video	Creator showed PowerPoint and explained science behind mindfulness, in person	Facilitators showed video of creator going through PowerPoint on smart screen in person	Facilitators shared video of creator going through PowerPoint through screen sharing on Zoom
Experiential video	Creator led participants in a mindfulness meditation with gentle yoga stretches, in person	Facilitators showed video of creator leading participants in a mindfulness meditation with gentle yoga stretches on smart screen in person	Facilitators shared video of creator leading participants in a mindfulness meditation with gentle yoga stretches through screen sharing on Zoom
Reflection questions	Completed as participants arrived in room, at beginning of each session		Sent out one day prior to session with the expectation that they would be completed prior to session; shared slide at beginning of session with reflection questions to refresh participants' memory
Breath counts	Completed and written in workbook page that was collected after program without identifying information		Completed and entered into anonymous Qualtrics link that was sent in Zoom chat
Continuing education credits	Paperwork completed after each session for nurses and social workers earning CE credits		Qualtrics survey created and survey link sent in Zoom chat to nurses and social workers earning CE credits at the end of each session
Facilitator and fidelity checker meetings	N/A (no facilitators and fidelity checkers)	Met Consistently	Met Consistently
Discussion questions	Open-ended standard discussion question (for specific week) with voluntary sharing, led by creator	Open-ended standard discussion question (for specific week) with voluntary sharing, led by facilitator	Modified to make questions more direct and succinct, voluntary sharing still led by facilitator
Music for Pavlovian effect	Music played as participants enter room and fill out reflection questions at start of session, as well as during experiential practice		Music shared by facilitator through audio share on Zoom while participants join meeting and during experiential practice
Home practices	Already fully virtual on website		

Comparison analyses of the major outcomes of burnout, resilience, perceived stress, and work engagement were conducted comparing Pre-COVID and COVID cohorts, in order to determine the reliability of virtual delivery when compared to traditional delivery of MIM. Analyses were independent samples *t*-tests run on the STATA/SE16 statistical software package.

To determine whether a HCP met burnout criteria, scores on the subscales of emotional exhaustion, depersonalization, and personal accomplishment of the Maslach Burnout Inventory (MBI) were analyzed (Maslach et al., 2010). A HCP was considered burnt-out if their score on the emotional exhaustion subscale was >27, if their score on the depersonalization subscale was >13, or if their score on the personal accomplishment subscale was lower than 31 (Maslach et al., 2001). Qualifying scores on one or more subscales indicated participant burnout.

## Results

### Demographic Data

Participants in the Pre-COVID cohort (*n* = 124) were 88% female, 11% male, and 1% other gender. They were 83% white, 6% black, 6% Asian, and 5% other race, and 5% identified as having Hispanic or Latino origins.

Participants in the COVID cohort (*n* = 99) were 84% female and 16% male. They were 88% white, 3% black, 7% Asian, and 2% other race, and 7% identified as having Hispanic or Latino origins.

### Quantitative Outcome Measures

**Figure 1:**
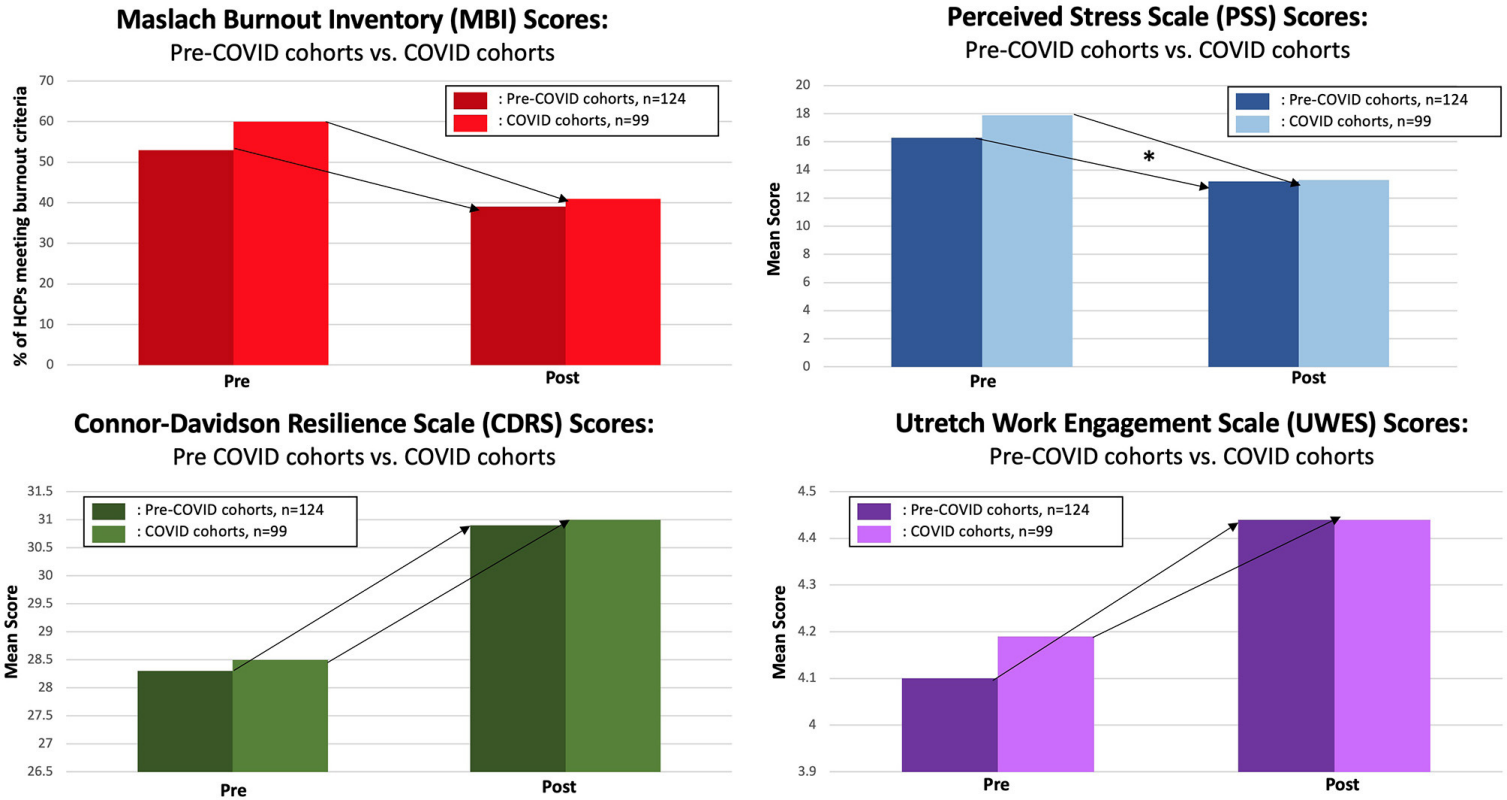
Pre-COVID vs. COVID cohort comparisons on burnout, perceived stress, resilience, and work engagement (*denotes significant *p*-value, *p* < 0.05).

**Figure 2:**
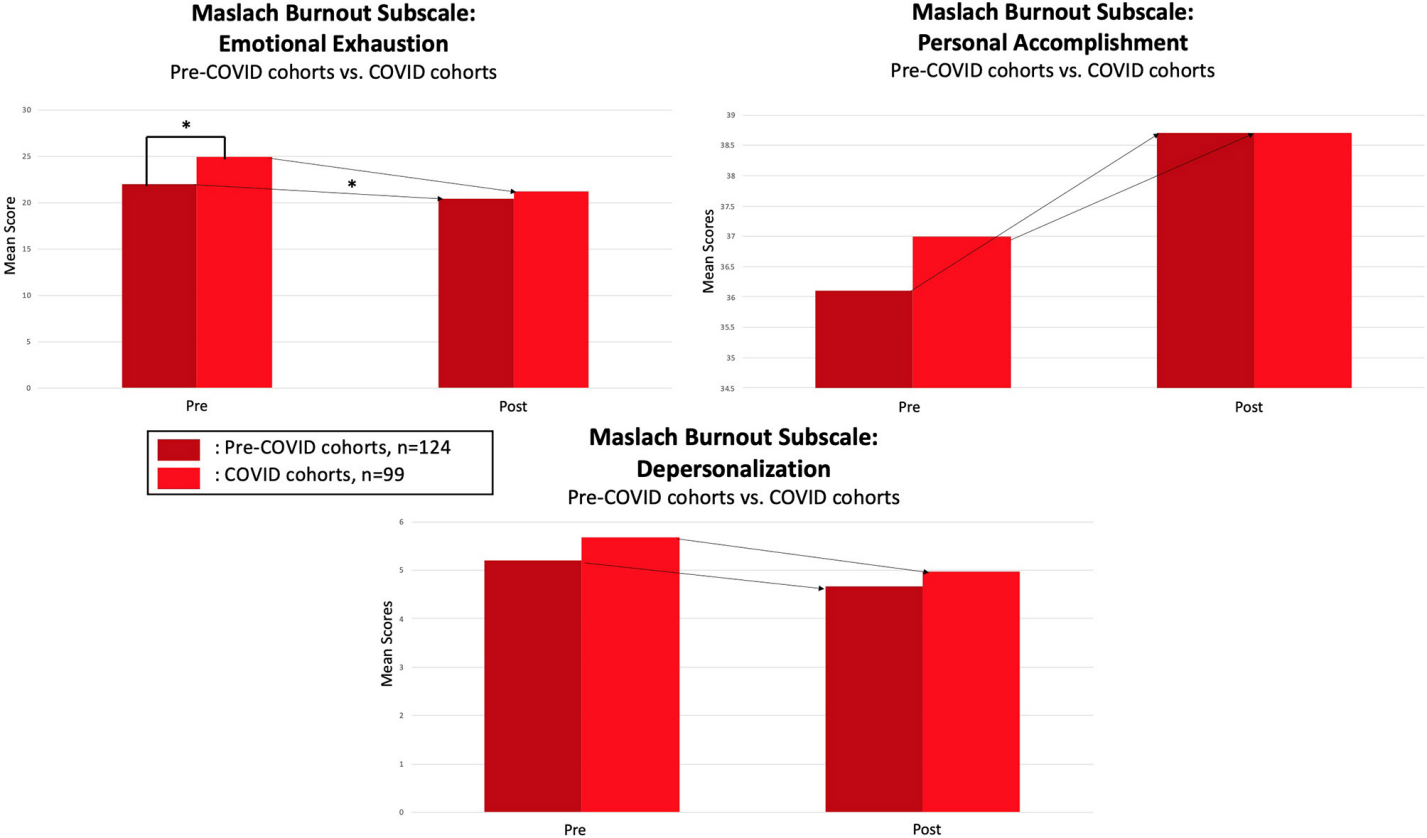
Pre-COVID vs, COVID cohort comparisons on MBI subscales (*denotes significant *p*-value, *p* < 0.05).

Analyses showed no significant differences between Pre-COVID and COVID cohorts in terms of the reduction in participants meeting burnout criteria by intervention end as compared to baseline (*p* = 0.2950) (see Figure 1). Although there was a non-significant difference in the reduction of participants meeting burnout criteria as determined by the Maslach Burnout Inventory, the subscale of emotional exhaustion showed a significant difference (*p* = 0.0315) with the COVID cohorts showing greater improvement (see Figure 2). COVID cohorts had a significantly higher mean emotional exhaustion baseline score when compared to Pre-COVID cohorts (*p* = 0.0361). The subscales of depersonalization and personal accomplishment showed no significant differences (*p* = 0.3876, *p* = 0.1519, respectively). Similarly, there were no significant differences in resilience scores as determined by the Connor Davidson Resilience Scale (CDRS) (*p* = 0.4259) (Connor and Davidson, 2003) or in work engagement scores as determined by the Utrecht Work Engagement Scale (UWES) (*p* = 0.1984) (Schaufeli et al., 2006). However, there was a significant difference in perceived stress scores as determined by the Perceived Stress Scale (PSS) (*p* = 0.0405), showing greater improvement than the Pre-COVID cohorts (Cohen et al., 1983). COVID cohorts had a significantly higher mean perceived stress baseline score when compared to Pre-COVID cohorts (*p* = 0.0255).

### Qualitative Outcomes

Qualitative analysis of participant responses to weekly reflection prompts in the COVID cohorts revealed community support as an integral benefit of MIM during the pandemic (Table 2).

**Table 2:** Qualitative feedback from COVID cohorts.

**Responses to weekly reflection prompts**	**Themes identified in virtual delivery of MIM**
“To see and hear others perspective elsewhere within the health system and use what I have learned to better my life within my career is huge!”	Community support gained from MIM
“Hopefully the culture will continue to be more mindful”	
“The discussion this week in class was great!”	
“Examples of how to incorporate wellness practices and mindfulness practices throughout my days and weeks, as opposed to only during set meditation times. The group sharing concrete experiences and practices helped foster this practice.”	
“Will use it daily with my students and patients”	Cultivating mindfulness in clinical practice/creating a mindful community in clinical practice
“Once I do this, I can approach the patient again from a clearer more empathetic place”	
“I have become aware of this over time and it has helped me in my relationships with my co-workers and participants in my research studies. I have learned not to react to negative comments and to treat all with genuine kindness. It has been amazing to see the change come over people when they are treated with respect and kindness. If we could all do this on a regular basis, what a kinder and gentler world this would be. Meditation is the answer!!”	
“I feel my patients are benefiting. I like to now pause and take a breath before I proceed to becoming susceptible to the stress of the environment”	
“Sanitizing my hands as I go in and out of patient rooms helps me to be more mindful as I go through my day.”	
‘When seeing patients with mental health concerns, I've discussed mindfulness, breathing, and staying present”	
“The most helpful thing is the ability to help remind myself to first think, feel, then respond. In day-to-day life as a healthcare provider, typically we are trained to respond and respond quickly. Although jumping through hoops and putting out fires isn't the best way to react. This program retrains the HCP to stop, think, feel (process), and then respond.”	Managing stress as a healthcare provider during COVID-19 hardships
“Taking some deep breaths at times of increased stress did help calm me down. And yes, my patients benefitted from having a calmer physician”	
“When feeling overwhelmed with questions and needs coming from each direction, I was able to listen to my own breath and bring myself to the moment”	
“I think that when I was able to understand that I do not have to take on someone else's sadness and frustrations but can still be empathetic. Some people have so many social and physical hardships. I can still help them and empathize without taking on all of their hardships”	
“The mindful sleep was surprisingly helpful, as I began having regular dreams for the first time in a decade. These dreams were a pleasant replacement of what in recent months has been the voices, faces, and eyes of dying COVID patients.”	
“This shift of separating the emotions from my identity allowed me better handle these emotions, but also enabled me to feel multiple things at once: ‘I am feeling grateful that COVID numbers are decreasing in our ICU but I am also feeling frustrated and disappointed in the people minimizing COVID or denying my experience. And that's okay.”'	

### Attendance Rates

Attendance rates were similar between Pre-COVID and COVID cohorts. The Pre-COVID cohorts' average attendance rate was 84%, and the COVID cohorts' average attendance rate was 80%.

## Discussion

The results presented highlight the effectiveness of virtual MIM programming, by showing the reliability of results as compared to Pre-COVID, in-person delivery of MIM. While burnout, resilience, and work engagement showed similar improvements when comparing pre-post MIM changes between Pre-COVID and COVID cohorts, there were significant differences in emotional exhaustion and perceived stress. This is reflected in the qualitative feedback as well, as participants often mentioned increased stress levels due to the demands and trauma of being an HCP during COVID-19. In addition, analyses show that the baseline levels of emotional exhaustion and perceived stress were in fact significantly higher for the COVID cohorts than the Pre-COVID cohorts. This indicates that significant differences in emotional exhaustion and perceived stress between COVID and Pre-COVID cohorts may be due to the intense stressful pandemic environment rather than the intervention delivery method. HCPs needed an intervention to help them through the unique stresses presented by the pandemic, and virtually delivered MIM proved effective in doing so. Synchronous virtual delivery was not expected to yield better results, but it did. This outcome makes sense given the high baseline scores of the COVID cohorts, and the timeliness of a resiliency building intervention. Furthermore, the non-significant differences in results between Pre-COVID and COVID cohorts show that MIM as an intervention is effective across varied levels of stressed HCP populations, regardless of delivery method. This indicates support for MIM being an intervention that is preventative and reactive; for the COVID cohorts, MIM was a reactive response to increased HCP stress, while for the Pre-COVID cohorts, MIM is a preventative response to the daily demands of being a HCP. In emailed feedback to the first author, various past participants expressed gratitude that they had experienced MIM prior to COVID, as they expressed that it ameliorated their stress response as HCPs during this time.

### Mindfulness Is Contagious: MIM Is Both Preventative and Reactive

Although analyses only confirm the effectiveness of MIM on its participants, trends within the data have shown its ability to create a mindful medical center (Klatt M. D. et al., 2020). Indication of this increasing mindfulness culture at The Ohio State University Wexner Medical Center (OSUWMC) may be seen in the baseline resiliency scores. Prior to the onset of the pandemic, over 420 HCPs at OSUWMC had completed this organizationally sponsored program, perhaps prompting a halo effect for others, extending benefits that go beyond the participant to others within the organization. With so many OSUWMC HCPs exposed to formal mindfulness programming, they have reported being more mindful with their patients, colleagues, and supervisors, eliciting a culture change at the medical center (Klatt M. D. et al., 2020). This could be a protective function of MIM in times of increased stress and demands of HCPs, such as during a pandemic. This may explain the higher baseline resiliency scores in COVID cohorts (Figure 1) while pointing to MIM's effectiveness across varied degrees of environmental stress. MIM has shown itself to be a preventative approach and perhaps a reactive measure to HCP stress, which was only exacerbated by COVID-19.

### Existing Virtual Mindfulness Programming

The results of the present study align with recent findings in the literature about effects of online mindfulness-based interventions. Multiple studies have investigated the effectiveness of online mindfulness training specifically among interprofessional healthcare professional populations, finding benefits in perceived stress, empathy, and resilience (Kemper and Khirallah, 2015; Kemper and Yun, 2015; Kemper, 2017). A noted limitation to these studies, however, was the lack of adherence, with only 68% of participants completing the full asynchronous mindfulness program (Kemper, 2017). Our study, in comparison, had similar attendance rates to in-person MIM, highlighting the potential benefits of synchronous online mindfulness offerings. The similarities in attendance rates of Pre-COVID and COVID cohorts provide support that MIM is feasible in both a fully virtual delivery model and an in-person delivery model. Attendance data reflects virtual delivery user experience was feasible and acceptable.

Other online mindfulness studies have examined populations for which in-person mindfulness is not an option, such as rural placement of medical students, large medical campuses where in-person attendance would take time to travel to the meeting site, or in the instance of a pandemic which warranted our study. In a 2020 study with rural medical student participants, results showed that half of the participants regularly practiced mindfulness meditation after the 8-week program, with 32% continuing to practice 4 months beyond intervention end. In addition, medical students' perceived stress levels significantly decreased while self-compassion levels significantly increased 4 months beyond intervention end (Moore et al., 2020). While other studies have shown immediate improvements following online mindfulness interventions, this study points to sustainable outcomes that can lead to a more mindful healthcare professional population (Klatt et al., 2021).

Further support of a synchronous structure comes from a meta-analysis of randomized controlled trials of online mindfulness, with results showing higher effect sizes for guided vs. unguided online mindfulness interventions. The effect sizes were higher for guided interventions in depression, anxiety, well-being, and perceived stress (Spijkerman et al., 2016). This may highlight the importance of synchronous, vs. asynchronous online delivery of mindfulness programming.

### Barriers to Virtual Mindfulness Programming

A possible barrier to effective online mindfulness interventions is lack of time and lack of knowledge, according to a 2016 study on mindfulness interventions for medical residents (Taylor et al., 2016). This points to a need for organizationally-sponsored virtual mindfulness programs such as MIM, which have the potential to create a culture of mindfulness within a health system. Indeed, a multi-center randomized controlled trial showed that an online mindfulness intervention can significantly reduce perceived stress and anxiety of medical students, when offered (Warnecke et al., 2011). Other barriers to virtual mindfulness interventions as identified by the literature include higher attrition rates than typical of in-person interventions (Kemper and Khirallah, 2015; Spijkerman et al., 2016; Bailey et al., 2018) and a lack of objective measures (Kemper and Khirallah, 2015). With high attendance rates for both Pre-COVID and COVID cohorts, attrition was minimal in our study. However, like most virtual mindfulness programs, only self-report measures were used to obtain results. Future research should attempt to gain objective results in addition to self-report results, such as physiological correlates of stress.

### Strengths and Limitations of the Present Study

The present study reports effectiveness of virtual delivery of MIM, however, there are some notable limitations. One limitation is the difficulty in measuring engagement with the program content. When delivered in-person, facilitators can visually measure engagement, yet for virtual delivery, it is difficult to tell if participants are fully attending to the program content. However, given that results were similar to in-person delivery for burnout, resilience, and work engagement, this suggests that participant engagement was similar across the two delivery modes. Video cameras were requested to be turned on for the weekly sessions in an attempt to engage participants in each MIM session. Another limitation to virtual delivery of MIM, as well as all virtual interventions, is difficulty for participants to find access to technology with video cameras, a quiet and private space to join the program, and reliable internet. Program facilitators also must anticipate various types of computers, smartphones, and tablets, and plan to accommodate for a range of participant technological abilities. This made virtual delivery more time intensive for the MIM program manager during the 8 weeks of delivery compared to in-person delivery, but allowed MIM be scaled and disseminated to HCPs during the pandemic when it was sorely needed. Finally, this study, with a combined sample size of *n* = 223, is the first of its kind yet results should be confirmed through future experimental studies of virtual MIM. This was a single-arm, non-randomized study; future studies should include a control group and randomization to confirm results.

A strength of this study is that it directly compares in-person delivery to virtual delivery of the same intervention. While much of the existing research on virtual mindfulness-based interventions report on the effectiveness of virtual programming, this study adds to the literature by maintaining consistency of program content to allow for direct comparison. In addition, a strength of the present study is the transition of MIM from creator-led to hybrid delivery to virtual delivery, while maintaining consistent content through fidelity monitoring, consistent results through comparison analyses, and sustainability of those results for creator-led and hybrid delivery (Klatt et al., 2021). Sustainability of results from virtual delivery will be examined in the future.

## Conclusion

This study presents results of an unexpected benefit of the COVID-19 pandemic: the ability to effectively scale MIM to be delivered in three different modes, ranging from fully in person to fully virtually. This addressed the need for HCPs to feel supported and empowered during the pandemic when they needed it most. This “proof of concept,” the effective synchronous virtual delivery of MIM for HCPs, will allow for multisite effectiveness studies of MIM in the future as a means to help ameliorate HCP stress, regardless of physical location. Converting MIM to a fully virtual delivery mode was an unexpected benefit of a most challenging time. This transformation to virtual delivery will enable the scaling of MIM to assist HCPs in the everyday stresses they face, pandemic and beyond. The delivery of MIM is funded by the Health system, to support the health and wellness of employees, with no cost incurred by the HCP.

## Data Availability Statement

The raw data supporting the conclusions of this article will be made available by the authors, without undue reservation.

## Ethics Statement

The studies involving human participants were reviewed and approved by The Social and Behavioral Institutional Review Board of The Ohio State University. The patients/participants provided their written informed consent to participate in this study.

## Author Contributions

MK, OG, and AB conceived and designed the study and drafted the study protocol. RB, OG, AW, and MK drafted the manuscript. MK provided background and rationale, along with key content related to results and discussion, and critically reviewed and revised the manuscript. AB and AW provided content on attendance and retention. OG and RB conducted the analyses. All authors reviewed and approved the final version of the manuscript.

## Conflict of Interest

The authors declare that the research was conducted in the absence of any commercial or financial relationships that could be construed as a potential conflict of interest.

## Publisher's Note

All claims expressed in this article are solely those of the authors and do not necessarily represent those of their affiliated organizations, or those of the publisher, the editors and the reviewers. Any product that may be evaluated in this article, or claim that may be made by its manufacturer, is not guaranteed or endorsed by the publisher.
